# 2D MR Spectroscopy Combined with Prior-Knowledge Fitting Is Sensitive to HCV-Associated Cerebral Metabolic Abnormalities

**DOI:** 10.1155/2012/179365

**Published:** 2012-07-12

**Authors:** Rajakumar Nagarajan, Manoj K. Sarma, April D. Thames, Steven A. Castellon, Charles H. Hinkin, M. Albert Thomas

**Affiliations:** ^1^Department of Radiological Sciences, David Geffen School of Medicine, University of California, Los Angeles, CA 90095-1721, USA; ^2^Department of Psychiatry and Biobehavioral Sciences, University of California, Los Angeles, CA 90095, USA; ^3^Greater Los Angeles VA Healthcare System, University of California, Los Angeles, CA 90073, USA

## Abstract

There is an evidence of neurocognitive dysfunction even in the absence of advanced liver disease in chronic hepatitis C virus (HCV) infection. Brain metabolism has been investigated non-invasively using one-dimensional (1D) *in vivo* Magnetic Resonance Spectroscopy (MRS) over three decades. Even though highly concentrated cerebral metabolites (N-acetylaspartate, creatine, choline, glutamate/glutamine, myo-inositol) have been detected using MRS, other metabolites at low concentrations (~1–3 mM or less) including glutathione, aspartate and GABA are quite difficult to observe using 1D MRS. In order to resolve overlapping resonances from a number of metabolites, a remedy is to add a second spectral dimension to the existing 1D MRS. Localized two-dimensional correlated spectroscopy (L-COSY) has been developed over the last decade to enhance the spectral dispersion by using the second spectral dimension. We have evaluated this L-COSY technique in the frontal white/gray matter regions of 14 HCV+ (mean age of 56.2 years) and 14 HCV− (mean age of 46.6 years) subjects. Our preliminary results showed significantly increased myo-inositol and glutathione in the HCV+ compared to the HCV− subjects. Hence, glutathione and myo-inositol should be considered along with other metabolites as important markers of inflammation.

## 1. Introduction

The natural course of Hepatitis C virus (HCV) infection, which is transmitted via parenteral route, is modulated by both host and viral factors and typically tends to evolve towards chronicity, which will ensue in approximately 85% of cases following acute infection [1]. Given that this virus affects approximately 3% of the world's population [2], HCV infection represents a leading cause of chronic hepatitis, cirrhosis, end-stage liver failure, and hepatocellular carcinoma. More recently, evidence suggests that HCV is capable of crossing the blood-brain barrier and that the brain serves as an important reservoir for subsequent viral replication [3–5].

 There is evidence that chronic HCV infection results in neurocognitive dysfunction even in the absence of advanced liver disease [6, 7]. In most cases, subtle cognitive complaints are reported that include difficulties in concentration and slowed processing speed. These observations sparked a number of investigations that sought to characterize neuropathological changes in patients with HCV with mild (noncirrhotic) liver disease.

*In vivo* proton (^1^H) magnetic resonance spectroscopy (MRS) is a noninvasive technique that gives information on brain metabolism at the end of a standard brain magnetic resonance imaging (MRI) protocol. Using proton MRS technique, Forton and colleagues were among the first groups to report cerebral metabolite abnormalities suggestive of frontal-subcortical dysfunction in patients with mild chronic HCV infection [8, 9]. Weissenborn et al. [10] used ^1^H MRS to study 30 HCV-infected patients with normal liver function who underwent cognitive testing. They found decreased N-acetylaspartate-to-creatine ratios (NAA/Cr) in the cerebral gray matter compared to healthy controls which can be attributed to either decreased NAA or increased Cr. NAA is a neuronal marker and Cr is a component of high-energy phosphate metabolism. There were no abnormalities in any other regions (parietooccipital white matter, basal ganglia, or pons) or any perturbations in choline containing compounds.

Among patients with noncirrhotic chronic hepatitis C, MRS has demonstrated altered cerebral metabolism in HCV [8, 10–12]. Elevated choline (Ch) and myoinositol (mI) ratios have been found in the basal ganglia, and central and frontal white matter of HCV-infected patients [11, 12]. McAndrews and colleagues performed ^1^H MRS analysis on their cohort of 37 HCV-infected patients with mild hepatitis [11]. Elevations in cerebral Ch and reductions in NAA were found in voxels that were localized to the central white matter. These findings are thought to reflect reduced neuronal integrity and inflammation or proliferation of glial cells.

Interestingly, the role of neurotransmitters such as *gamma*-aminobutyric acid (GABA), an inhibitory neurotransmitter, and glutathione (GSH), an antioxidant, has received less attention in the literature although GABA is essential for mental concentration and focus whereas GSH protects the brain against oxidative stress [13]. It is plausible that GABA and GSH may play an important role in HCV that is not detectable using conventional one-dimensional (1D) MRS spectral techniques.

 In 1D spectral representation, the overlap of metabolite peaks is caused by many features, including J-modulation leading to varying phase artifacts with echo time, TE. Hence, metabolite quantitation becomes an uphill task for many coupled metabolites. 1D MR spectral editing techniques to unravel the overlapping resonances rely on J-coupled proton metabolites that have well-separated multiplets. A technique based on subtraction methodology is very sensitive to motion artifacts leading to subtraction errors. An additional drawback is that only one metabolite can be identified at a time. Two-dimensional (2D) localized correlated spectroscopy (L-COSY) [14] overcomes this problem by adding a second frequency dimension to each spectrum by acquiring multiple 1D spectra with incrementally longer TEs and applying double Fourier transform on the set of spectra to produce a 2D spectrum. A hypothesis of this current study was that using a 2D MRS approach combined with ProFit quantitation would facilitate observing changes in cerebral metabolites such as NAA, choline groups, mI, GSH, and GABA more accurately than the previously reported 1D MRS work demonstrating the underlying central nervous system involvement in HCV. The goal of the present study was to employ an emerging MRS approach—2D L-COSY and combine that with a prior knowledge fitting (ProFit) algorithm [15, 16] to better characterize and quantify cerebral metabolite abnormalities present in HCV+ patients versus healthy controls.

## 2. Materials and Methods

Fourteen patients with advanced HCV disease (mean age of 56.2 years) and fourteen healthy controls (mean age of 46.6 years) were recruited from infectious disease clinics for the MRI/MRS study. All the scans were performed with a Siemens 3T Trio-Tim (Siemens Medical Solutions, Erlangen, Germany) MRI/MRS scanner using 12 channel head receive coil. The entire protocol was approved by the institutional review board (IRB), and informed consent was obtained from each human subject. A T1-weighted MRI procedure was used to guide to select a volume of tissue from which the 2D MRS were acquired. A WET (water suppression enhanced through T1 effects) method with three frequency-selective radiofrequency (RF) pulses was used for the water suppression [17]. The fast automatic shimming technique by mapping along projections (FASTESTMAP) [18] has been successfully used in order to get better line width. The water line width was ~15 Hz obtained in gray/white matter in the left frontal.

 For the 2D MRS, a 2D L-COSY sequence consisting three slice-selective RF pulses was used similar to 1D PRESS, but the last 180° pulse was replaced by 90° for the volume localization, with the last 90° RF pulse also enabling the coherence transfer necessary for 2D L-COSY. The spectral encoding for the second dimension was inserted between the second and third slice-selective pulses. 2D L-COSY spectra were recorded using the following parameters: effective echo time (TE) = 30 ms, repetition time (TR) = 2000 ms, and the total number of scans of 800 (100 Δ*t*_1_ increments and 8 averages per Δ*t*_1_), with voxel size of 3 × 3 × 3 cm^3^ corresponding to a total duration of approximately 26 min. The 2D raw matrix consisted of 2048 complex points along the first dimension and 100 points along the second dimension.

 The ProFit algorithm has been further optimized for the quantitation of 2D L-COSY. The ProFit algorithm was implemented using MATLAB (MathWorks, Natick, MA, USA, version 7.3) and was executed on an Intel 2.8 GHz with Windows XP. The ProFit algorithm uses prior knowledge constraints and a combined linear and nonlinear optimization for fitting. It uses a prior knowledge basis set generated using the GAMMA library [19] in combination with the chemical shift and J-coupling values reported in the literature [20].

 The ProFit algorithm quantified the following metabolites: creatine (Cr), N-acetylaspartate (NAA), glycerylphosphorylcholine (GPC), phosphorylcholine (PCh), free choline (Ch), alanine (Ala), aspartate (Asp), GABA, glucose (Glc), glutamine (Gln), glutamate (Glu), glycine (Gly), glutathione (GSH), lactate (Lac), myoinositol (mI), N-acetylaspartylglutamate (NAAG), phosphoethanolamine (PE), taurine (Tau), scyllo-inositol (Scy), and ascorbate (Asc). 2D L-COSY spectra were then processed using the modified ProFit code, and the measurement accuracy was characterized using Cramér-Rao lower bound (CRLB) [21].

 To avoid a false attribution of signals, five independent macromolecules (valine, leucine, isoleucine, threonine, and lysine) and three lipids (palmitic acid, linoleic acid, and oleic acid) were added with metabolites basis set in the ProFit algorithm. All the lipids basis were broadened by the Lorentzian filter.

 The metabolite differences between HCV+ and healthy controls were tested using a two-tailed *t*-test. A *P* value < 0.05 was considered statistically significant.

## 3. Results

**Figure 1:**
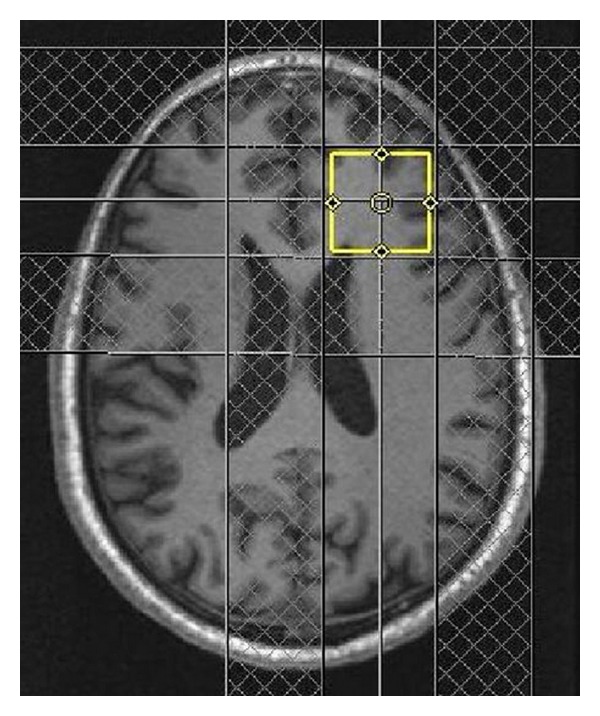
The axial MRI with MRS voxel location of a 64 years old HCV+ patient.

**Figure 2:**
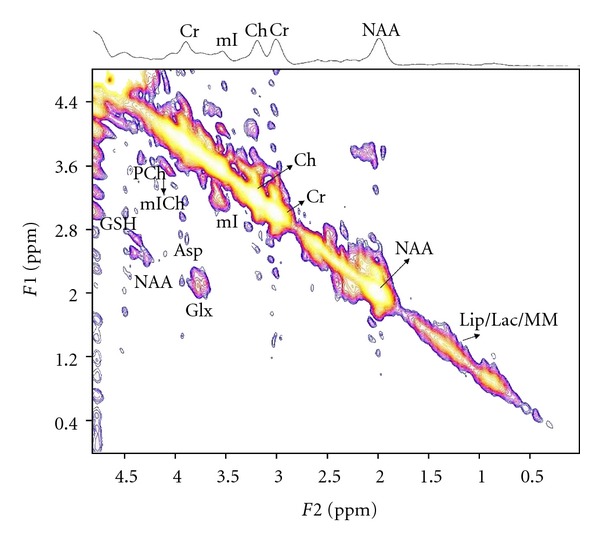
A representative 2D L-COSY spectrum of a 64 years old HCV+ patient.

**Figure 3:**
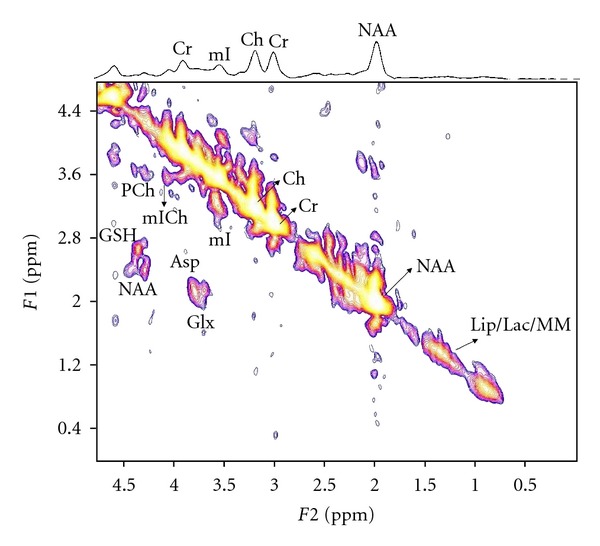
A representative 2D L-COSY spectrum of a 54 years old healthy control.

**Figure 4:**
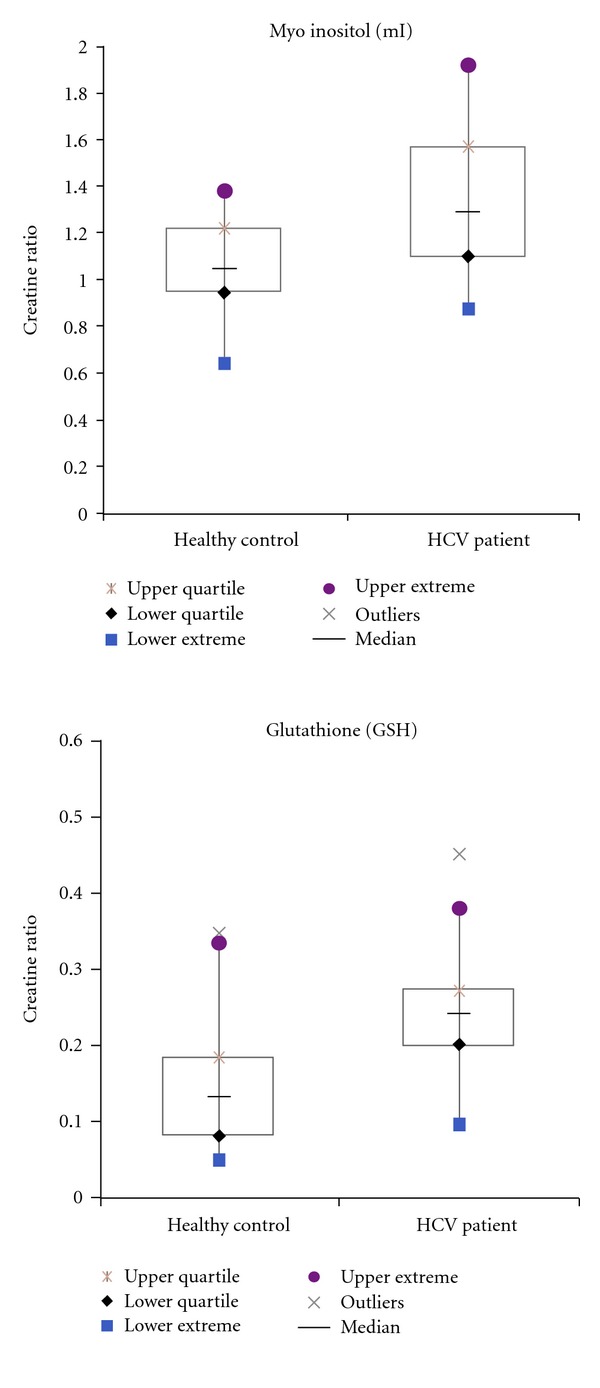
Box and Whisker plots of mI and GSH between healthy controls and HCV+ patients.

**Figure 5:**
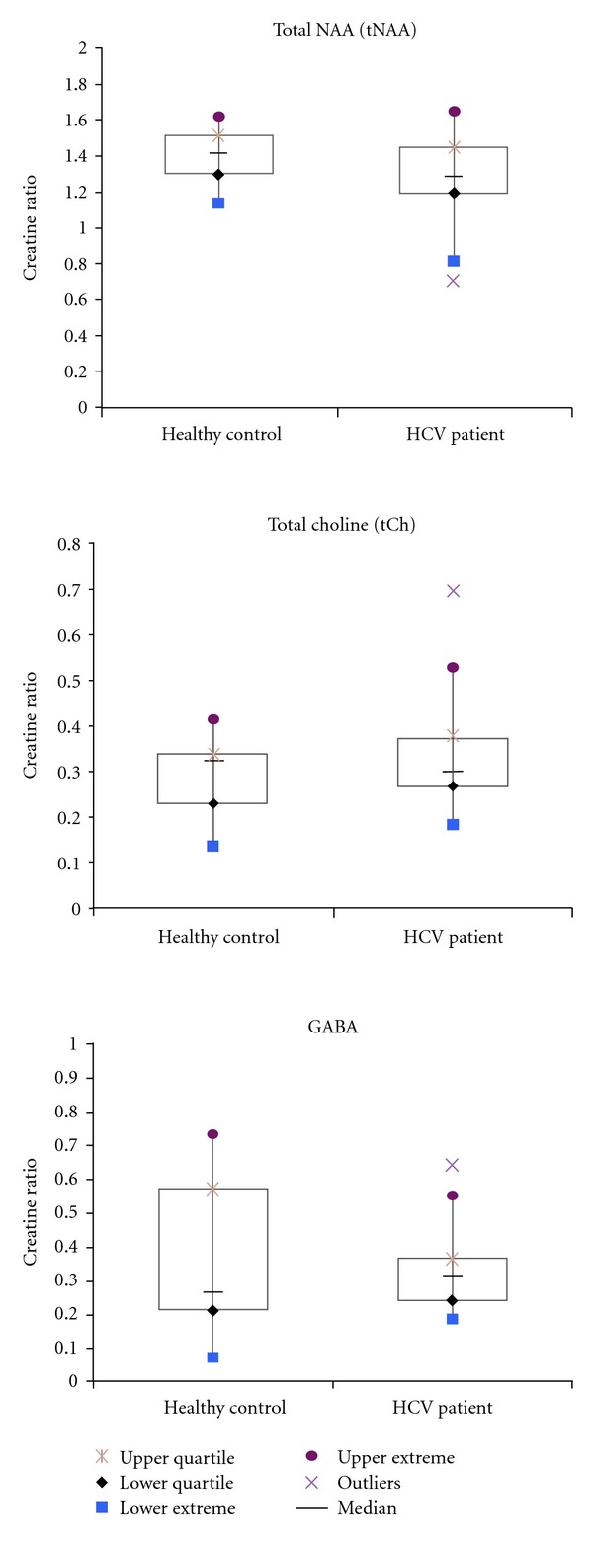
Box and Whisker plots of selected metabolites changes between healthy controls and HCV+ patients.

Figure 1 shows the MRS voxel location in the prefrontal white mater on the axial MRI of a 64 years old HCV+ patient. Figures 2 and 3 show 2D L-COSY spectra of a 64 years old HCV+ patient and a 54 years old healthy control, respectively, recorded in the same voxel location shown in Figure 1.  Table 1 shows the mean and standard deviation (SD) of selected metabolite concentrations with respect to Cr of healthy controls and HCV+ patients.  Figure 4 shows the Box and Whisker plots of mI and GSH between healthy controls and HCV+ patients.  Figure 5 shows Box and Whisker plots of selected metabolites changes between healthy controls and HCV+ patients.

 Significantly increased GSH (*P* = 0.003) and mI (*P* = 0.029) with respect to Cr were observed in HCV+ patients compared to healthy controls as shown in Figure 4. There were no significant differences observed in any other metabolite ratios (Table 1) even though there was a trend of decreased total NAA (tNAA = NAA + NAAG) and GABA and increased total choline (tCh = GPC + PCh + Ch) in the patients' group.

## 4. Discussion

Our results showed significant increase of mI and GSH in the HCV+ patient group compared to healthy controls and demonstrated that the 2D MRS spectra allowed us to visualize and characterize the role of GSH in cerebral metabolism. Elevation of mI levels was also consistent with prior studies that have indicated mI as an important marker of inflammation and glial proliferation among patients with neuroinflammatory disorders [22]. Myoinositol is found only in glial cells and is also a constituent of membrane lipids [23]. Increased levels are believed to reflect glial cell activation and increased cell membrane turnover [24, 25]. Elevations in the mI/Cr and decreased NAA/Cr ratios have been identified using ^1^H-MRS in HIV-infected patients [26–28].

 GSH is a tripeptide that serves as a major antioxidant and vital component of host defenses. Its primary role is to protect tissues from free radical injury via detoxification and repair of injury [29]. The concentration of GSH in human brain is in the range of 1–3 mM and can exist intracellularly in either an oxidized (GSSG) or reduced (GSH) form. Glutathione is an important antioxidant and plays a role in the detoxification of electrophilic compounds and peroxides via catalysis by glutathione-S-transferases (GST) and glutathione peroxidases (GPx) [30]. Maintaining optimal GSH : GSSH ratios in the cell is critical to survival. An increased GSSG-to-GSH ratio is considered indicative of oxidative stress. Oxidative stress generated by the production of reaction oxygen species (ROS) appears to be connected with the loss of neurons during the progression of neurodegenerative diseases. Evidence suggests that GSH plays an important role in the detoxification of ROS in the brain [13]. Our results suggest that elevations in GSH are indicative of oxidative stress in those with HCV infection, which is consistent with the elevations in mI. Considering that we did not observe statistically significant differences in NAA between patients and controls, it is possible that GSH is an early marker of inflammation that precedes actual neuronal damage. Elevations in GSH levels may occur relatively early after HCV crosses the blood-brain barrier. Hence, GSH should be considered along with other metabolites as an important marker of inflammation.

 A trend of elevated Ch was observed in the patient group compared to healthy controls. Elevated levels of Ch reflect increased cell membrane turnover and have also been reported in other neuroinflammatory conditions [22, 31]. Choline increase has been observed in the presence of macrophage infiltration of the brain, for example, in patients with HIV infection with and without AIDS dementia complex [32, 33] and in other chronic infections such as the John Cunningham (JC) virus infection [34] or subacute sclerosing panencephalitis [35]. Choline and mI are also putative markers for glial cell inflammation, and activation and elevations are believed to reflect cellular proliferation due to infection or inflammation [9, 11]. Immune cell activation by macrophages and/or neuronal astrocytes has been shown to produce Ch peaks in HIV patients.

 While there was a trend towards lower levels of NAA and GABA in the HCV+ group, this was not statistically significant. GABA serves as the primary inhibitory neurotransmitter at 20% to 44% of cortical neurons [36, 37]. Changes in GABA metabolism may play an important role in the origin and spread of seizure activity [37–39]. Several reports suggest that GABAergic neurons are decreased in the epileptic neocortex [37, 40–42]. Significant reductions in cerebrospinal fluid (CSF) GABA concentration are seen in patients with various epileptic syndromes [43].

## 5. Conclusions

Our preliminary results showing increased mI and Ch and decreased NAA are in agreement with previous reports [8–10]. Additional findings from this work include the quantitation of GSH, GABA, Glu, Scy, and Asp using the 2D L-COSY spectra postprocessed by the ProFit algorithm. Elevation in GSH may represent oxidative stress in the brain. Future studies should employ longitudinal methods to better characterize metabolic changes that occur as a result of HCV infection and determine whether elevations in GSH precede significant decreases in NAA. Understanding the attendant changes in cerebral metabolism will allow us to understand how HCV treatments alter the course of neurological functioning. The ability to detect early abnormal changes in cerebral metabolism among HCV patients is useful for healthcare providers working with HCV-infected patients, as they will be aware of the nature and course of cognitive changes that occur during the course of the disease. These preliminary results need to be reproduced in a larger cohort of HCV+ and HCV− subjects.

## Figures and Tables

**Table 1:** Metabolite changes between healthy controls and HCV+ patients calculated from the 2D L-COSY data.

Metabolites/Cr	Healthy controls (mean ± SD) *n* = 14	HCV+ patients (mean ± SD) *n* = 14
tNAA	1.405 ± 0.153	1.303 ± 0.253
tCh	0.291 ± 0.092	0.331 ± 0.126
Glu+Gln	1.838 ± 0.310	1.780 ± 0.582
mI^†^	1.056 ± 0.237	1.311 ± 0.264
Scy	0.089 ± 0.049	0.094 ± 0.035
GSH^†^	0.140 ± 0.040	0.243 ± 0.090
Glu	1.585 ± 0.447	1.631 ± 0.546
GABA	0.371 ± 0.226	0.331 ± 0.137
Asp	0.398 ± 0.070	0.417 ± 0.080
GPC	0.166 ± 0.074	0.176 ± 0.047

^†^ *P* value < 0.05.
